# Supplementary material for: Content-rich biological network constructed by mining PubMed abstracts
Source: BMC Bioinformatics. 2004 Oct 8;5:147. doi: 10.1186/1471-2105-5-147 (PMC528731; doi:10.1186/1471-2105-5-147)
Supplement: Additional File 2 — The original results of the above study (non-essential files are deleted to keep the file size under the limit set by BMC bioinformatics). [file 1471-2105-5-147-S2.bz2 › chilibotAdditionalFile2/dip05/202ID8652557E691/html/ERCC2_ERCC5.html]

 


 **ERCC2** and **ERCC5** 
  
Found 28 abstracts in PubMed, retrieved 05.  
 

 What does Google say? 
 PDF only 
| .edu only 

---

**Interactive relationship** (e.g. stimulation, inhibition, etc)

**Non-interactive relationship** (e.g. studied together, co-existance, homology, etc.)

- However, cells deficient in the other NER genes, XPD  [ **ERCC2** ] , XPB and XPG  [ **ERCC5** ] , all have the same ratio of exchanges to breaks as wild type.  Ref: 12509251 DNA Repair Amst , 2002
- In contrast to cells bearing mutations in other components of the nucleotide excision repair apparatus XPB, XPD  [ **ERCC2** ] , XPG  [ **ERCC5** ]  and CSB, cells defective for the ERCC1 XPF structure specific nuclease are highly sensitive to cisplatin.  Ref: 12202770 Nucleic Acids Res, 2002
- We have compared the frequency of polymorphisms in the NER genes, XPD  [ **ERCC2** ] , XPF, XPG  [ **ERCC5** ] , ERCC1.  Ref: 12865926 Br J Cancer, 2003
